# Supplementary material for: Therapeutic Effect of Biomimetic Scaffold Loaded with Human Amniotic Epithelial Cell-Derived Neural-like Cells for Spinal Cord Injury
Source: Bioengineering (Basel). 2022 Oct 9;9(10):535. doi: 10.3390/bioengineering9100535 (PMC9598945; doi:10.3390/bioengineering9100535)
Supplement: Supplementary file 1 [file bioengineering-09-00535-s001.zip › bioengineering-1857195-supplementary.pdf]

Supplementary Materials

Biomimetic spinal cord scaffold loaded with human amniotic epithelial cells-derived neural-like cells repairs spinal cord injury in rats

Chen Qiu [1,2,†], Yuan Sun [3,†], Jinying Li [1,2], Yuchen Xu [4,5,6], Jiayi Zhou [1,2], Cong Qiu [1,2], Shaomin Zhang [4,5,6], Yong He [3,*] and Luyang Yu [1,2,*]

1. MOE Laboratory of Biosystems Homeostasis & Protection of College of Life Sciences, Zhejiang University, Hangzhou 310027, China
2. College of Life Sciences-iCell Biotechnology Regenerative Biomedicine Laboratory, Joint Research Centre for Engineering Biology, Zhejiang University-University of Edinburgh Institute, Zhejiang University, Haining 314400, China
3. Key Laboratory of 3D Printing Process and Equipment of Zhejiang Province, School of Mechanical Engineering, Zhejiang University, Hangzhou 310027, China
4. Qiushi Academy for Advanced Studies, Zhejiang University, Hangzhou 310027, China
5. Key Laboratory of Biomedical Engineering of Ministry of Education, Zhejiang University, Hangzhou 310027, China
6. Zhejiang Provincial Key Laboratory of Cardio-Cerebral Vascular Detection Technology and Medicinal Effectiveness Appraisal, Zhejiang University, Hangzhou 310027, China

* Correspondence: yongqin@zju.edu.cn (Y.H.); luyangyu@zju.edu.cn (L.Y.)

† These authors contributed equally.

Table S1. Primers used for qRT-PCR in this study.

Genes	Forward Primer (5′-3′)	Reverse Primer (5′-3′)
GAPDH	CATTGCCCTCAACGACCACTTTGT	TCTCTCTCTTCCTCTTGTGCTCTTGC
Pax6	GGCAACCTACGCAAGATGGC	TGAGGGCTGTGTCTGTTCGG
Sox2	CATGCACCGCTACGACG	CCCTGGAGTGGGAGGAAGA
Olig2	TGGCTTCAAGTCATCCTCGTC	CCAGTCGCTTCATCTCCTCC
Nkx6.1	ACACGAGACCCACTTTTTCCG	TGCTGGACTTGTGCTTCTTCAAC
NeuroD1	ATGACCAAATCGTACAGCGAG	GTTCATGGCTTCGAGGTCGT
Neurofilament (NF-H)	ATTCCTTTCTCGCTTCCAG	CAGACTTCTCCACCACTTTGAT
Otx2	CAAAGTGAGACCTGCCAAAAAGA	TGGACAAGGGATCTGACAGTG
Map2	CCCCTTGCTTCCCTGTAGAA	ATTTCCTCCTGGCAACCTCA
NeuN	TTCTATGCAGTGACGGGGTT	TCCATCCTGATACACGACCG

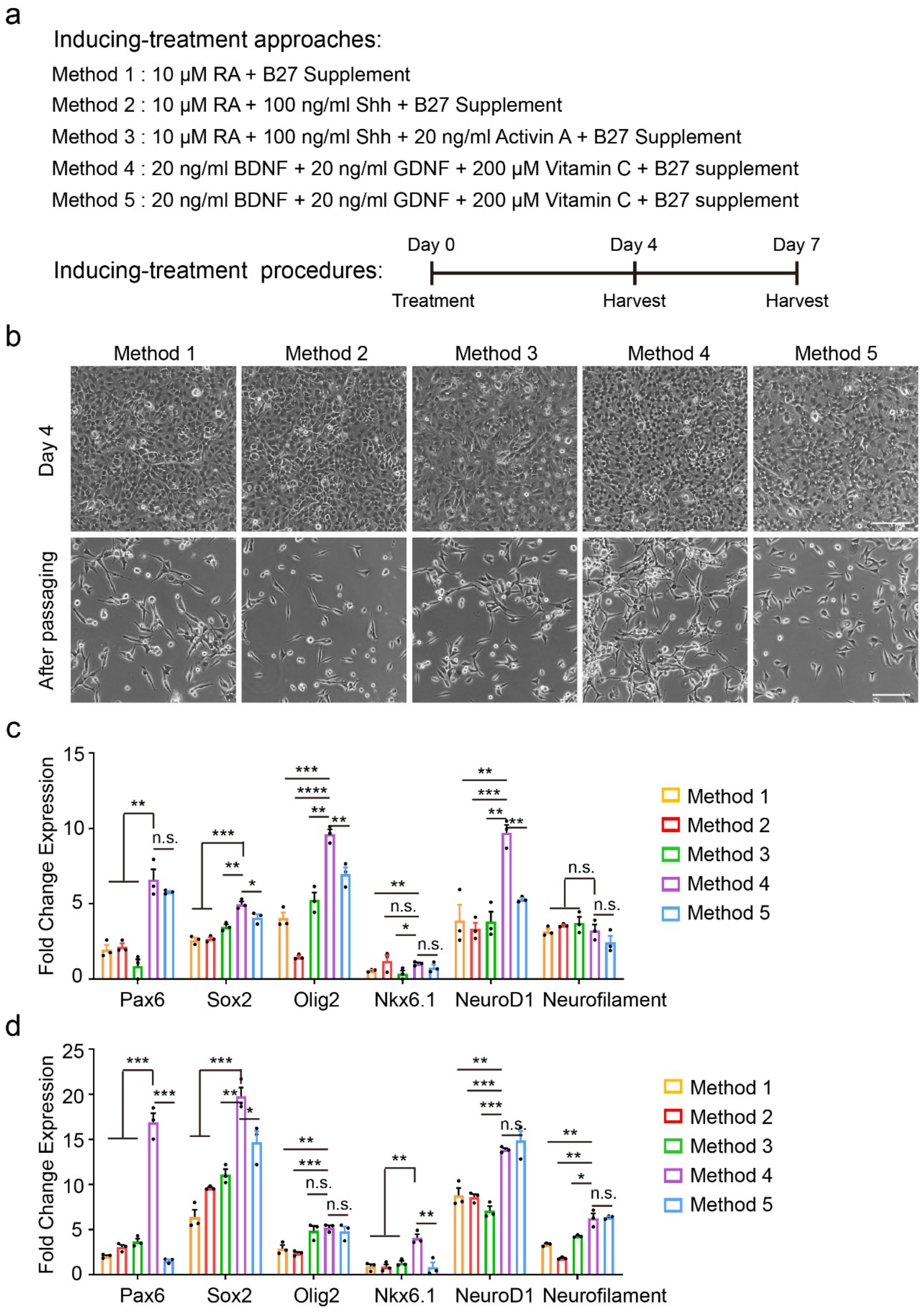

Figure S1. The screening of inducing-treatment approaches. (**a**) Experimental schematic of 5 different inducing-treatment approaches; (**b**) The observation of cells morphology from indicated treated groups at 4 days and after passaging; (**c**) The mRNA expression of neural markers from all indicated treated groups 4 days after inducing-treatment, normalize the non-treated hAECs as 1, ****$p<0.0001$, ***$p<0.001$, **$p<0.005$, *$p<0.05$, n.s.: not significant (Unpaired

t-test), n=3; (**d**) The mRNA expression of neural markers from all indicated treated groups 7 days after treatment, normalize the non-treated hAECs as 1, ****p<0.0001, ***p<0.001, **p<0.005, *p<0.05, n.s.: not significant (Unpaired t-test), n=3. **Scale bars: b:** 100 μm.

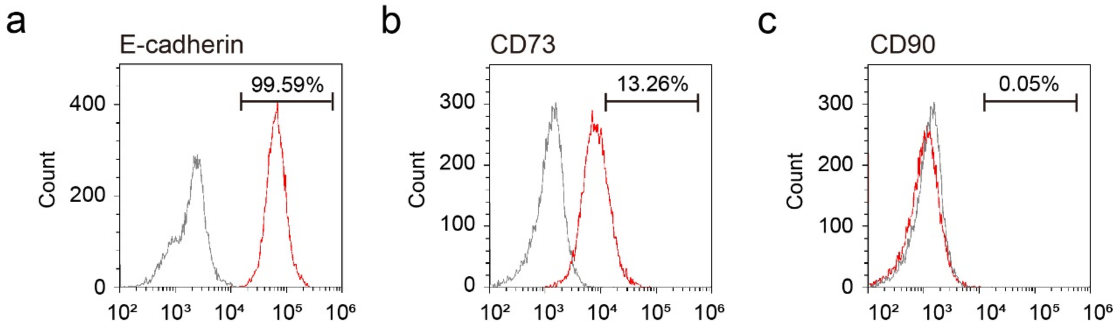

Figure S2. The detection of epithelial and mesenchymal properties of hAEC-neural-like cells. (**a-c**) The expression of epithelial marker (E-cadherin) and mesenchymal markers (CD73, CD90) detected by flow cytometry after BGVB inducing treatment.

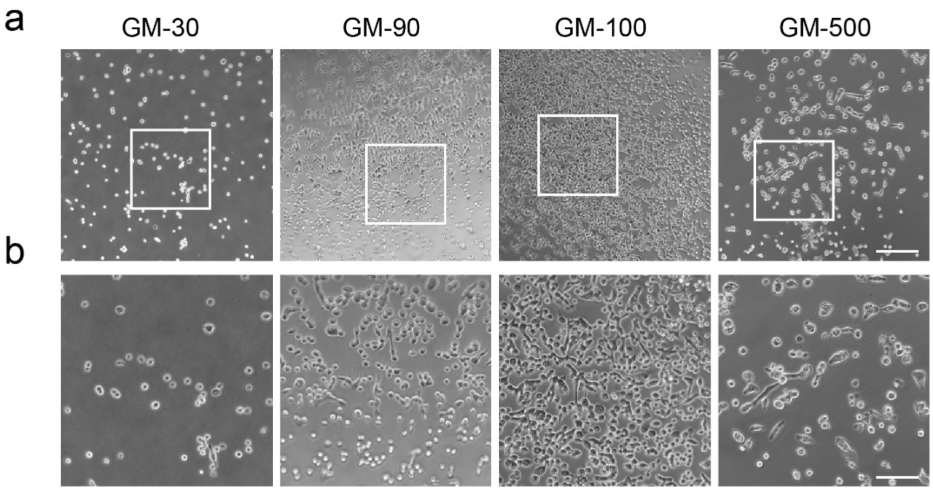

Figure S3. The optimization of GelMA substitution degree for hAECs attachment and growth. (**a**) The morphology of hAECs on GelMA surfaces with 4 different substitution degrees; (**b**) The magnified imagings of the white frames in **figure a** from all indicated GelMA surfaces showed the significant difference in cell number and appereance. **Scale bars: a:** 100 μm; **b:** 50 μm.

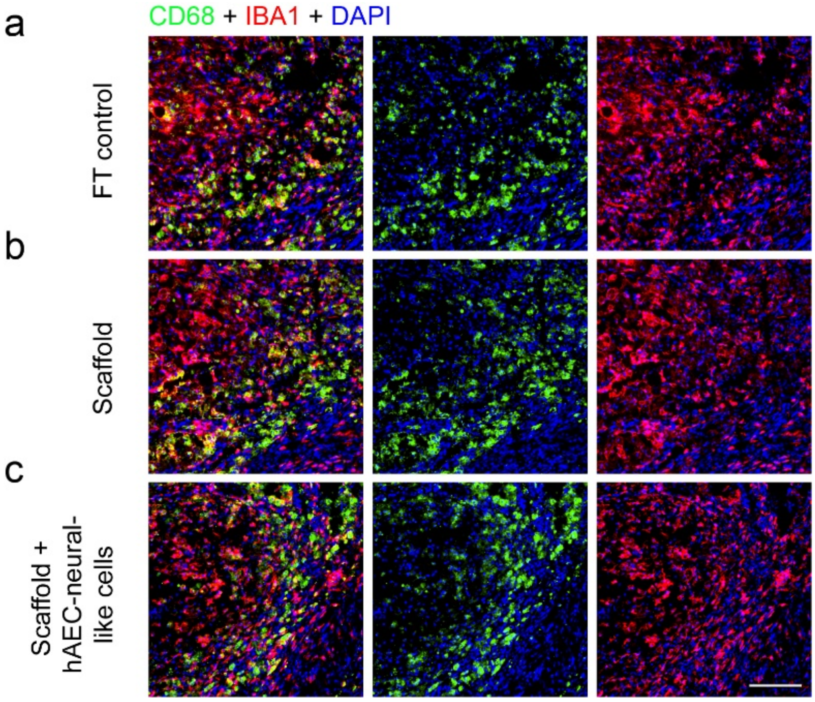

Figure S4. The evaluation of inflammation response around lesions from all indicated groups. (**a-c**) CD68 (green) and IBA1 (red) staining revealed the activation of macrophage and microglia around lesion from different treatment groups; (**a**) The FT control group; (**b**) The empty scaffold group; (**c**) The scaffold + hAEC-neural-like cells group. **Scale bars:** 140 μm.
